# Women in National Life

**Published:** 1921-10

**Authors:** E. M. Field


### WOMEN IN NATIONAL LIFE.

By Mrs. E. M. Field.

Towards the end of September large newspaper placards in the city of Sheffield bore the inscription, "What *do* women want?" Those who listened to the discussions of six to seven hundred delegates from federated Societies and local branches of the National Council of Women got a simple answer. What the vast mass of thinking women want is to serve their country, and, in order to do so efficiently, to educate themselves to a real understanding of its problems. For, as the Bishop of Sheffield happily said, "every problem of to-day has a history and a need of scientific treatment." The Council, earlier known as the National Union of Women Workers, grew up in the latter half of the last century, when single workers joined together in societies, and societies presently found a need for cohesion and mutual aid to save waste and overlapping, and failure through limitation of scope. Practically every society of any importance is now affiliated, the College of Nursing amongst others. And the British Council is linked with corresponding organisations in some thirty other countries, Jugo-Slavia and Portugal being recent recruits. An International Conference takes place every five years: Norway received the last gathering. The English Council meets yearly in some inviting centre. Workers combine for special purposes on Committees, which are really smaller Councils, to discuss and forward a policy in public health, industry, emigration, and so on.

Taken as a whole, the present aim of the Council, as shown in discussion of resolutions and of addresses by special speakers, is to work for the solution of our most pressing social problems through efforts for the better protection of children and adolescents, and a better education throughout the whole nation. It is interesting to note the value ascribed to hospital training and to a medical education for women. In giving her experience of V.A.D. work, Lady Ampthill said that the greatest need of the girls who offered service in numbers seemed to be, first, some standard, not necessarily a high one, but definite, of general education, and, secondly, the trained ability to do some one thing well. These the trained nurse possesses, but time and material had to be deplorably wasted by others who owned merely to a taste for music and drawing and a supposed power of organisation, but who knitted badly, sewed unpractically, thought cookery a menial service, and knew no foreign language adequately. Professor Winifred Cullis went further and urged that a medical training, even for a girl who had no intention of practising, would be priceless in rendering her an efficient citizen.

No resolution was so vigorously debated as one which urged an increased teaching of citizenship in secondary schools and training colleges, this last so that some beginning might be made in elementary schools. Our universities have now erected social science into a diploma subject, and junior branches of the National Council itself are being formed in many places, so that girls and women can inform themselves. All this may be spade-work, but upon it certainly depends the soundness of work done for the moral and physical health of the nation.

The Council's Public Health Committee have passed a resolution that instruction upon venereal diseases shall form a part of every nurse's training. But the Council holds that the evil of which these are the outcome needs to be attacked by women's efforts nearer to its root. At Sheffield they passed resolutions tending to remove the obstacles to women's work on juries, and heard from Professor Cullis a plea for their entrance into the professions of solicitor and barrister. The women J.P.s belonging to the Council—a majority of those appointed—have formed a Committee of their own for study. It is hoped that by the more established presence of women in Courts such a scandal as the recent appearance of a child of six alone amongst men for the trial of an assault case will become impossible. The Council wishes to see the age for Children's Courts raised to seventeen, the application of probation where possible, and in any case the avoidance of prison, which turns girls into prostitutes and lads too often into criminals. They will further legislation for the longer protection of girls, and it is interesting in this connection to note that the recent dismissal of two policewomen at Plymouth as superfluous has had the unexpected result of bringing forward women candidates for the Town Council. Again, a resolution earnestly spoken to by a member of the Actors' Union demands a licence, at present only required in London, for managers of theatrical companies. Bogus managers have been responsible for many additions to the sad categories of unmarried mothers and unfathered children. Another resolution asked for the extension of Trade Boards. These Boards have been severely criticised, especially for over-much raising of wages for mere learners. But they have disposed of those starvation wages which have been one cause at least of casual prostitution and consequent venereal disease.

An "urgent" resolution asked for the representation of women, by a woman doctor if possible, on the Health Committee of the League of Nations, where at present thirteen medical men have seats.

---

The recent visit paid by English physicians to the spas of France and Switzerland, suggests that it might well be succeeded by a visit to English spas from the physicians of those countries. Such a visit would make the home spas better known, even to English doctors. At present the home spa is neglected by the home doctor. Thus at the annual meeting of the Devonshire Hospital and Buxton Bath Charity, Dr. C. W. Buckley, the honorary physician, declared that the result of its research work was far too little appreciated. No other institution of the same size, he went on, was devoted to the study of rheumatic disease, and every method worth testing was tried with care. The proper use of the Buxton mineral waters was a good method, but the literature on rheumatic treatment made little mention of them.

